# Substituted pteridinones, pyrimidines, pyrrolopyrimidines, and purines as p90 ribosomal S6 protein kinase-2 (RSK2) inhibitors: Pharmacophore modeling data

**DOI:** 10.1016/j.dib.2021.107433

**Published:** 2021-09-25

**Authors:** Becka M. Warfield, Kimberly A. Casalvieri, Christopher J. Matheson, Donald S. Backos, Philip Reigan

**Affiliations:** Department of Pharmaceutical Sciences, Skaggs School of Pharmacy and Pharmaceutical Sciences, University of Colorado Anschutz Medical Campus, 12850 East Montview Boulevard, Aurora, CO 80045, United States

**Keywords:** RSK2, Kinase, Inhibitor, Pharmacophore model, Structure-activity relationship

## Abstract

The RSK2 kinase is a downstream effector of the Ras/Raf/MEK/ERK pathway that is aberrantly active in a range of cancer types and has been recognized an anticancer target. The inhibition of RSK2 kinase activity would disrupt multiple pro-cancer processes; however, there are few RSK2 inhibitors. The data have been obtained for a series of pteridinone-, pyrimidine-, purine-, and pyrrolopyrimidine-based compounds, developed to establish a structure-activity relationship for RSK inhibition. The compounds were docked into the ATP-binding site of the N-terminal domain of the RSK2 kinase using Glide. The binding conformations of these molecules was then used to generate a set of pharmacophore models to determine the structural requirements for RSK2 inhibition. Through the combination of these models, common features (pharmacophores) can be identified that can inform the development of further small molecule RSK inhibitors. The synthesis and evaluation of the pteridinone- and pyrimidine-based compounds was reported in the related articles: Substituted pteridinones as p90 ribosomal S6 protein kinase (RSK) inhibitors: A structure-activity study (Casalvieri et al., 2020) and Molecular docking of substituted pteridinones and pyrimidines to the ATP-binding site of the N-terminal domain of RSK2 and associated MM/GBSA and molecular field datasets (Casalvieri et al., 2020). [1], [2]. The synthesis and evaluation of the purine- and pyrrolopyrimidine-based compounds was reported in the related research article: N-substituted pyrrolopyrimidines and purines as p90 ribosomal S6 protein kinase-2 (RSK2) inhibitors (Casalvieri et al., 2021) [3].

**Specifications Table**

**Table:** 

Subject	Computer Graphics and Computer-Aided Design
Specific subject area	Computational-based pharmacophore modeling
Type of data	Images, figures
How data were acquired	PerkinElmer ChemDraw Prime, Schrodinger Maestro 12.5 (Release 2020-3) Glide and Phase
Data format	Raw, analyzed, and filtered
Parameters for data collection	Pharmacophore models were generated using experimentally determined IC_50_ values for previously docked (and aligned) compounds. Default feature settings and Phase hypo scoring were used for model generation.
Description of data collection	Pharmacophore models for previously docked (and aligned) BI-D1870-, purine-, and pyrrolopyrimidine-based compounds were generated using Phase through the calculation of vector, volume, site scores, survival scores, and survival activities. The Supplementary Material contains two separate project files generated by Schrodinger containing all parameters and raw data associated with protein and ligand preparation, Glide XP docking, and Phase pharmacophore modeling for BI-D1870-, purine-, and pyrrolopyrimidine-based compounds.
Data source location	Institution: University of Colorado City/Town/Region: Aurora, Colorado 80045 Country: USA Latitude: 39° 44′ 25.41″ N; Longitude: -104° 50′ 9.47″ W
Data accessibility	Data is with this article
Related research article	K. A. Casalvieri, C. J. Matheson, D. S. Backos, P Reigan. Substituted pteridinones as p90 ribosomal S6 protein kinase (RSK) inhibitors: A structure-activity study. Bioorg Med Chem. 2020; 28(5):115303. DOI: 10.1016/j.bmc.2019.115303. K.A. Casalvieri, C.J. Matheson, B.M. Warfield, D.S. Backos, P. Reigan, *N*-Substituted pyrrolopyrimidines and purines as p90 ribosomal S6 protein kinase-2 (RSK2) inhibitors, Bioorg. Med. Chem. 2021; 41:116220. https://doi.org/10.1016/j.bmc.2021.116220.

## Value of the Data

• These pharmacophore models were generated based on data from 2 compound sets and included known commercially available pan-RSK inhibitors of nanomolar potency and a selection of 35 pteridinone-, pyrimidine-, purine-, and pyrrolopyrimidine-based compounds with varying potencies of RSK2 inhibitory activity.
• The models summarize the data of 2 structure-activity studies for RSK2 inhibition and were generated to rationalize IC_50_ values obtained from purified recombinant RSK2 activity assays and support a model of the structure-activity relationship for these compounds detailed in the related articles.
• The fused heterocyclic rings of the pteridinone-, purine-, and pyrrolopyrimidine-based compounds may not be essential and “ring-opened” substitutions at C-5 and C-6 could be explored in future work.
• The pharmacophore modeling data generated supports a structure-activity relationship around RSK2 inhibition, informing not only of favorable combinations of electronic and steric features responsible for inhibition, but also of the structural features ideal for positioning in the ATP-binding site.
• The pharmacophore modeling data can be used to inform subsequent RSK inhibitor development and additional data may refine the model further to establish a pharmacophore model for RSK isoform-selective targeting.

## 1 Data Description

**Fig. 1:**
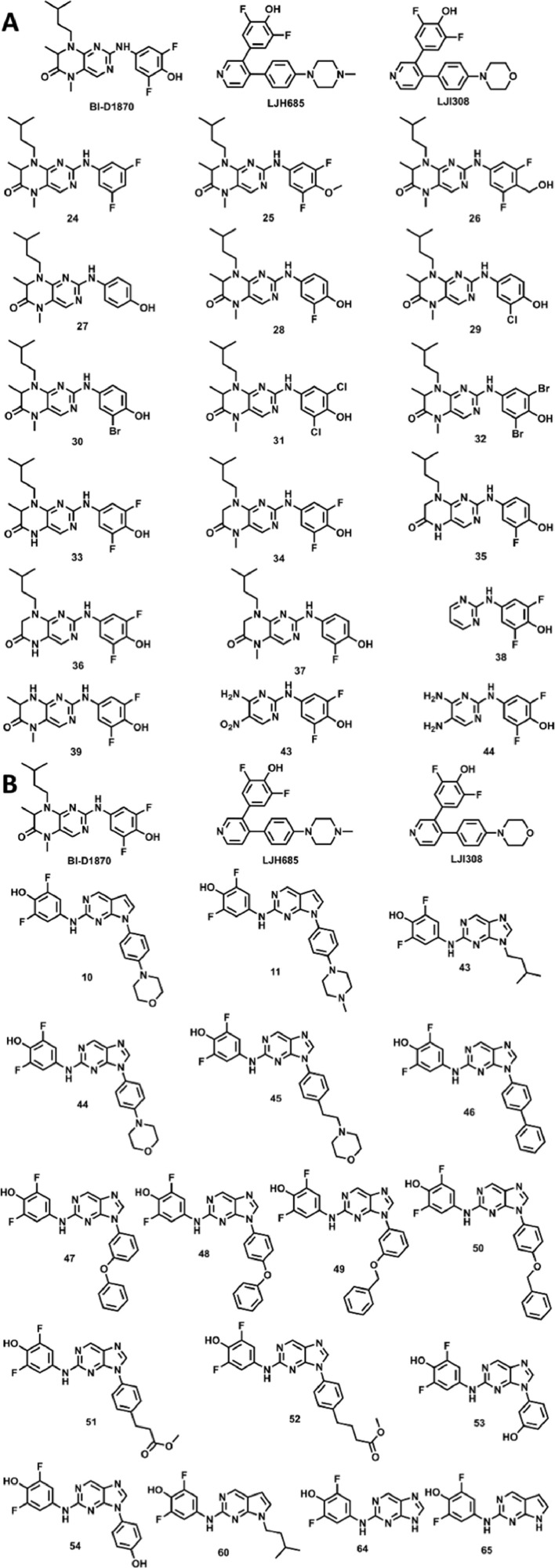
Chemical structures of A) pteridinone- and pyrimidine-based (numbering retained from Casalvieri, et. al., 2020) and B) purine- and pyrrolopyrimidine-based compounds (numbering retained from Casalvieri, et. al., 2021) docked into the ATP-binding site of the RSK2 NTKD.

**Fig. 2:**
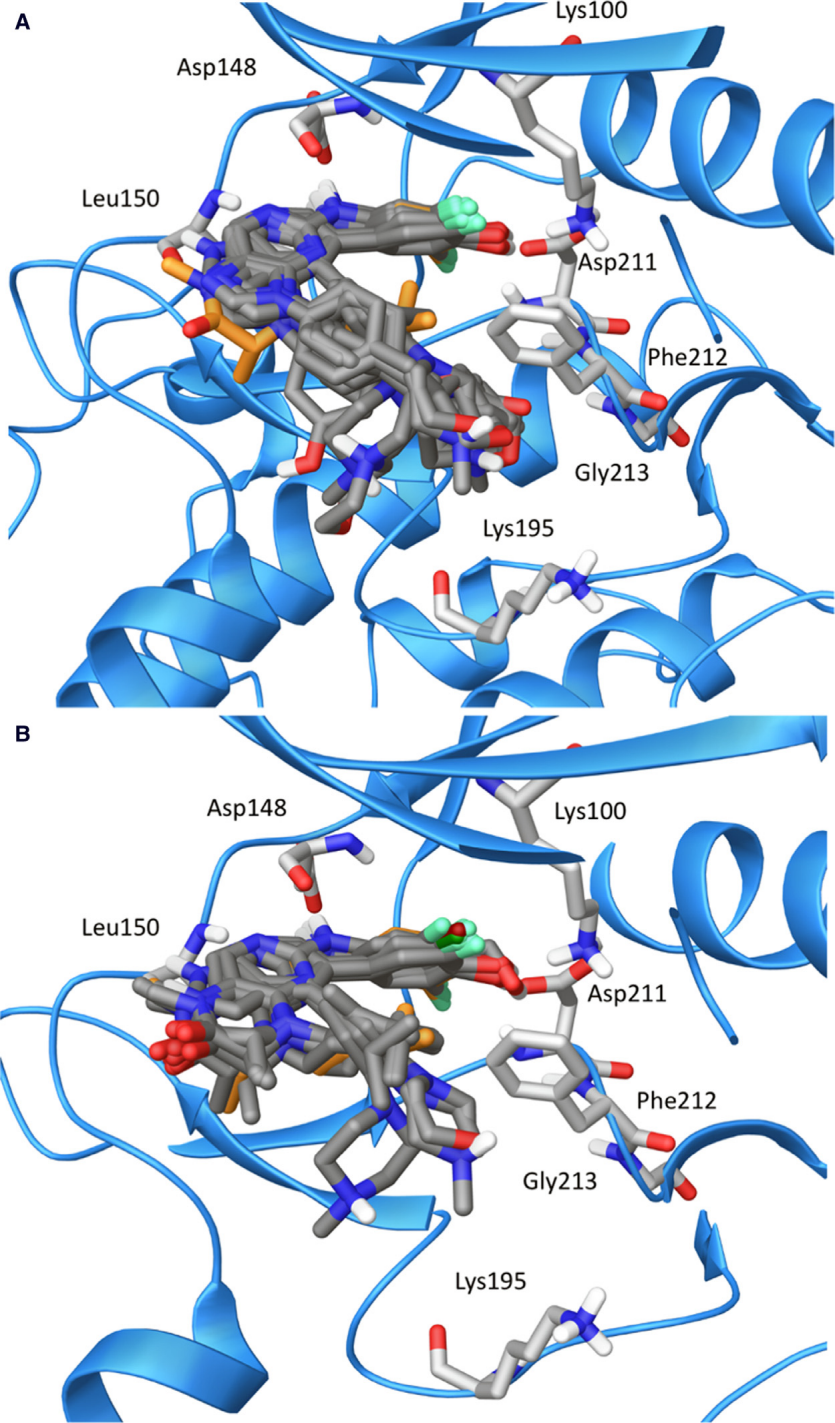
Overlays of the docked conformations of active matches for the top-ranking pharmacophore hypotheses as determined by Phase. A) 14 BI-D1870-based compounds (carbons colored dark gray), B) 17 purine- and pyrrolopyrimidine-based compounds in the ATP-binding site of RSK2 NTKD (blue ribbon representation) with stick display style representation of amino acid residues (carbons colored light gray), and BI-D1870 docked for reference in orange.

**Fig. 3:**
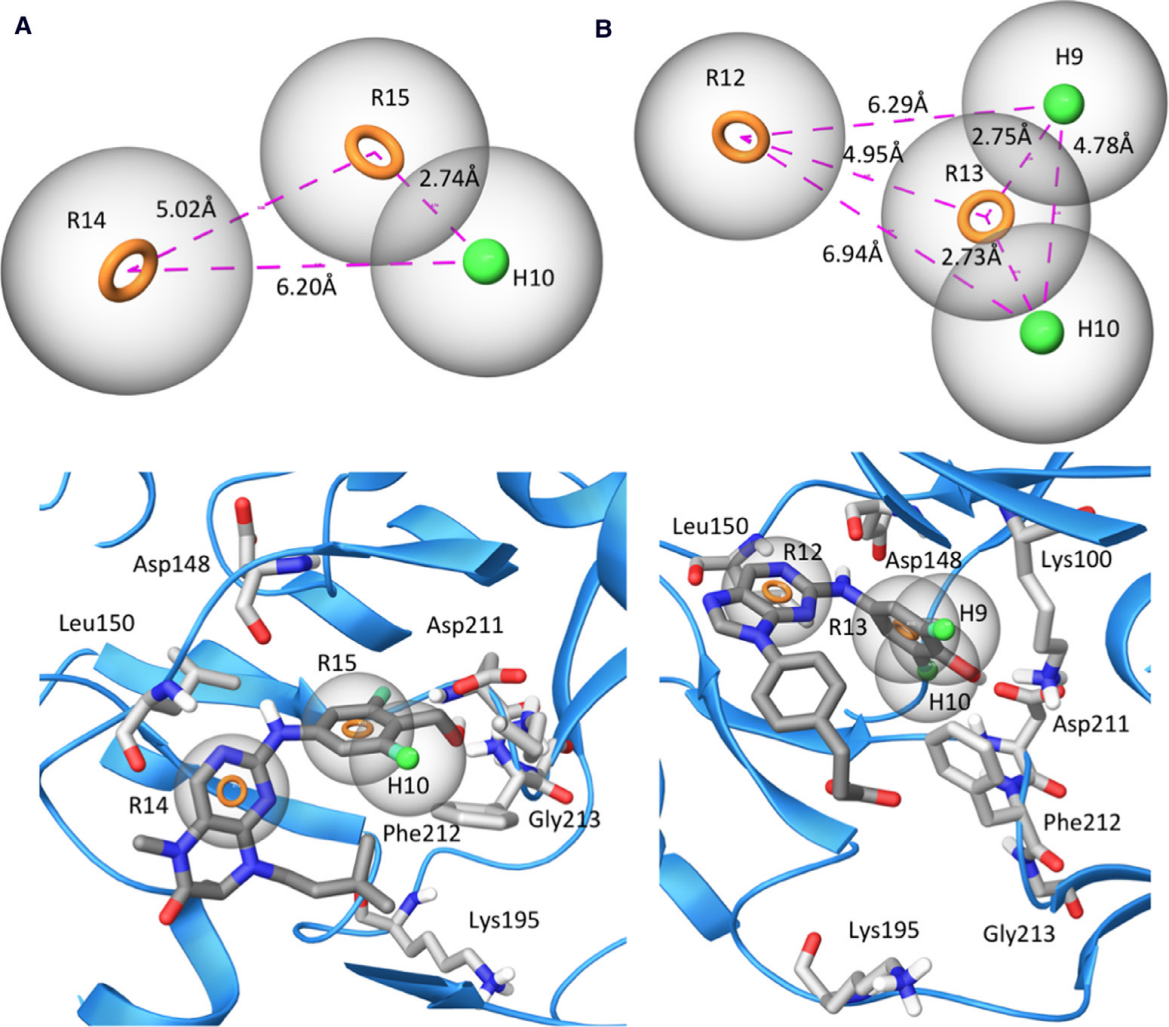
Pharmacophore model A) HHR_2 based on BI-D1870 as the reference ligand (carbons colored dark gray) and B) HHRR_1 based on 52 as the reference ligand (carbons colored dark gray) docked in the ATP-binding site of the RSK2 NTKD (blue ribbons with amino acid carbons in light gray), where feature tolerances are gray spheres, aromatic rings are orange rings (R) and green spheres are hydrophobes (H).

Ribosomal S6 protein kinase-2 (RSK2) is a member of the highly conserved RSK family of Ser/Thr kinases (RSK1-4) previously identified as targets in cancer [4]. RSK2 plays roles in the regulation of various cellular processes such as cell transformation and proliferation and the maintenance of cancer stem cells (CSCs) [4]. Several pan-RSK inhibitors have been developed to target the catalytic N-terminal kinase domain (NTKD) and the activating C-terminal kinase domain (CTKD) [4]; however, there are currently no isoform-selective inhibitors due to the high degree of sequence homology shared by the RSK family members. BI-D1870 is the “gold-standard” small molecule pan-RSK inhibitor, that targets the ATP-binding site of the NTKD and has been used to elucidate RSK function in cells [5]. Although BI-D1870 is potent pan-RSK inhibitor, it's use as a clinical anticancer agent is limited due to its poor pharmacokinetic (PK) properties [6,7]. This prompted our group to perform a structure-activity study of BI-D1870 to determine the structural requirements for RSK2 inhibition and reveal potential avenues for modification to improve PK properties [1], [2]. In addition, we also examined a series of N-substituted purines and pyrrolopyrimidines to probe the area outside the ATP-binding site that may provide opportunity for isoform-selective inhibitor development. Therefore, two distinct series of compounds were iteratively developed to understand the required structural features for RSK2 inhibition (Fig. 1), and computational modeling guided the design of these compounds and was used to determine critical interactions. Here, we provide computational-based pharmacophore models, their parameters and outputs for pteridinone-, pyrimidine, purine-, and pyrrolopyrimidine-based compounds evaluated in our analysis (Fig. 1, Fig. 2, Fig. 3). Our data support that there is opportunity for more optimized side chains that extend from the ATP-binding site and compliment the residues of the neighboring RSK2 substrate cavity. The Supplementary Material contains two separate project files generated by Schrodinger containing all parameters and raw data associated with protein and ligand preparation, Glide XP docking, and Phase pharmacophore modeling for BI-D1870-, purine-, and pyrrolopyrimidine-based compounds. The Supplementary Material contains two separate project files generated by Schrodinger containing all parameters and raw data associated with protein and ligand preparation, Glide XP docking, and Phase pharmacophore modeling for BI-D1870-, purine-, and pyrrolopyrimidine-based compounds.

## 2 Experimental Design, Materials and Methods

### 2.1 Molecular docking

The Glide module of Schrödinger (Release 2020-3, Schrödinger LLC, New York, NY, https://www.schrodinger.com) was used to dock both the series of pteridinone-, pyrimidine-, purine-, and pyrrolopyrimidine-based compounds (Fig. 1), including the known pan-RSK inhibitors BI-D1870, LJH685, and KJI308, into the ATP-binding site of the N-terminal kinase domain (NTKD) of the RSK2 (PDB: 4NUS) crystal structure. Prior to docking, the water molecules were removed, and the protein was prepared by assigning bond orders, adding hydrogens, and repairing any side chains or missing amino acid sequences. Disulfide bonds were not created, but all other settings in the Protein Preparation Wizard were default. To complete protein preparation a restrained minimization of the protein structure was performed using the default constraint of 0.30Å RMSD and the OPLS_2005 force field [8]. The prepared proteins were subjected to SiteMap analysis [9], to identify top-ranked potential binding sites. Default features of SiteMap were used, requiring at least 15 site points per reported site, reporting up to 5 sites, and using a more restrictive hydrophobicity. Recognized sites from the analysis were cropped at 4Å from the nearest site point. SiteMap analysis identified the ATP-binding site in the NTKD. Docking grids were generated using Receptor Grid Generation by picking a point in the ATP-binding site generated from SiteMap analysis. All other settings for the Receptor Grid Generation were default, with no constraints, rotatable groups, or excluded volumes defined. BI-D1870 and analogs were prepared using LigPrep by generating possible states at the target pH 7.0 ± 2.0 using Epik. Ligands were desalted and tautomers were generated. Specified chiralities of stereoisomers were retained and, at most, 32 stereoisomers were generated per ligand. Finally, ligands were minimized by applying the OPLS_2005 force field [8]. Molecular docking simulations were performed using the Glide ligand docking module in XP (extra precision) mode with default settings and features, and included post-docking minimization [10].

### 2.2 Pharmacophore modeling

The Phase module of Schrödinger (Release 2020-3, Schrödinger LLC, New York, NY, https://www.schrodinger.com) [11,12] was used to develop pharmacophore models for pteridinone-, pyrimidine, purine-, and pyrrolopyrimidine-based compounds. The top-ranked docked conformation of each compound was imported to compose a set of pre-aligned ligands. The experimentally-determined IC_50_ value of each compound was included in order to generate pharmacophore hypotheses. Pteridinone- and pyrimidine-based compounds were taken as one series and purine- and pyrrolopyrimidine-based compounds as a second series, resulting in two distinct sets of pharmacophore hypotheses generated. The known pan-RSK inhibitors BI-D1870, LJH685, and KJI308 were included in both models. Inactive compounds were defined as any compound for which the recombinant kinase assay was unable to accurately determine an IC_50_ value, while compounds possessing accurately determined IC_50_ values experimentally were defined as active. Hypothesis generation was performed using default values and feature settings. Ranking of compounds and hypotheses was completed using the Phase hypo scoring function. Phase scored and ranked possible feature combinations able to produce common pharmacophores through the calculation of vector, volume, site scores, survival scores, and survival activities [11,12]. For the pteridinone- and pyrimidine-based compounds, Phase scored and ranked 10 possible feature combinations able to produce common pharmacophores. HHR_2, based on BI-D1870 as the reference ligand, was determined to be one of two high-ranking hypotheses. HHR_2 consists of two hydrophobes and one aromatic ring. For the purine- and pyrrolopyrimidine-based compounds, Phase scored and ranked 20 possible feature combinations able to produce common pharmacophores. HHRR_1, based on the *N*-substituted purine ester, **52**, as the reference ligand, was determined to be the highest ranking hypothesis. HHRR_1 consists of two aromatic rings and two hydrophobes.

## CRediT Author Statement

**Becka M. Warfield:** Conceptualization, Methodology, Data Curation, Writing – Original draft preparation; **Kimberly A. Casalvieri:** Investigation, Data Curation; **Christopher J. Matheson:** Investigation; **Donald S. Backos:** Supervision; **Philip Reigan:** Conceptualization, Writing – Review and Editing, Supervision.

## Declaration of Competing Interest

The authors declare that they have no known competing financial interests or personal relationships which have or could be perceived to have influenced the work reported in this article.

## Appendix Supplementary materials

Image, application 1
